# An Application of Deep Learning to Tactile Data for Object Recognition under Visual Guidance †

**DOI:** 10.3390/s19071534

**Published:** 2019-03-29

**Authors:** Ghazal Rouhafzay, Ana-Maria Cretu

**Affiliations:** Department of Systems and Computer Engineering, Carleton University, Ottawa, ON K1S 5B6, Canada; acretu@sce.carleton.ca

**Keywords:** Haptic exploration, visual attention, visuo-haptic interaction, tactile object recognition, Convolutional Neural Network

## Abstract

Drawing inspiration from haptic exploration of objects by humans, the current work proposes a novel framework for robotic tactile object recognition, where visual information in the form of a set of visually interesting points is employed to guide the process of tactile data acquisition. Neuroscience research confirms the integration of cutaneous data as a response to surface changes sensed by humans with data from joints, muscles, and bones (kinesthetic cues) for object recognition. On the other hand, psychological studies demonstrate that humans tend to follow object contours to perceive their global shape, which leads to object recognition. In compliance with these findings, a series of contours are determined around a set of 24 virtual objects from which bimodal tactile data (kinesthetic and cutaneous) are obtained sequentially and by adaptively changing the size of the sensor surface according to the object geometry for each object. A virtual Force Sensing Resistor array (FSR) is employed to capture cutaneous cues. Two different methods for sequential data classification are then implemented using Convolutional Neural Networks (CNN) and conventional classifiers, including support vector machines and k-nearest neighbors. In the case of conventional classifiers, we exploit contourlet transformation to extract features from tactile images. In the case of CNN, two networks are trained for cutaneous and kinesthetic data and a novel hybrid decision-making strategy is proposed for object recognition. The proposed framework is tested both for contours determined blindly (randomly determined contours of objects) and contours determined using a model of visual attention. Trained classifiers are tested on 4560 new sequential tactile data and the CNN trained over tactile data from object contours selected by the model of visual attention yields an accuracy of 98.97% which is the highest accuracy among other implemented approaches.

## 1. Introduction

As the second decade of the 21st century is ending, artificial intelligence and machine learning solutions are finding their place more and more in daily life. Besides the wide application of machine learning in data processing, planning, and decision-making, intelligent machines and robots are expected to perform all the physical tasks that humans do. These robots are required to reproduce human capabilities to be reliable substitutes for them. To achieve this, a huge research effort is devoted to both discover human brain functions and develop computational models to align technology on biological patterns. As such, the creation of artificial sense of touch is an important topic of interest. Many different tactile sensors are nowadays designed, produced, and available on the market [1]. However, data acquisition and processing techniques that make their use viable in practical applications are still required to evolve toward higher levels of efficiency.

In the case of human tactile perception, neuroscientists have revealed six different exploratory procedures that humans are make use of in order to perceive a stimulus by touch, among which “contour following” and “enclosure” are used for object recognition as they help exploring the shape of objects [2]. Klatsky et al. [3] mentioned the contribution of two sensory modalities in tactile perception, namely, cutaneous and kinesthetic cues. Cutaneous cues sensed by mechanoreceptors in the skin can obtain information about the texture, roughness, vibration, and temperature of a surface, while kinesthetic cues are provided by joints, bones, and muscles and supply information about the weight or the object’s shape [3].

On the other hand, haptic exploration of objects is believed to be more reliable when visual data is available [4]. Moreover, Amedi et al. [5] refer to multimodal cells in human brain responding to both visual and tactile data, concluding the close contribution of human visual and tactile systems.

With inspiration from the visuo-haptic contribution in human sensorial loop and in accordance with the contour following strategy that humans use for object recognition, in this work we simulate the process of tactile data acquisition from a dataset of 3D models where the tactile data includes both of the two tactile sensory modalities employed by humans (cutaneous and kinesthetic). To align the process of tactile data acquisition with reality, an adaptive procedure is introduced to reduce the size of sensor while increasing the spatial resolution to probe the locations where a real sensor of a determined size will not be able to acquire data due to geometrical features. A similar probing strategy is also used in humans when using fingertips to touch finer details of objects and the palm for larger surfaces. The acquired sequential data from object contours are then used for the purpose of object recognition. A computational model of visual attention (i.e., a biologically inspired model selecting relevant areas in a visual scene for further exploration and analysis, as in human visual system) is advantageously employed to guide the process of contour following and results are compared to the case where visual information is not available (blind contour following in Section 5). The originality of the work with respect to the literature is found, more specifically, in the technique we propose to employ visual data for object recognition.

The main contributions of the work are as follows. (1) Simulation of tactile images (cutaneous cues) using a virtual tactile sensor relying on working principle of Force Sensing Resistor (FSR) arrays. (2) Adaptive simulation of tactile images according to geometry of objects. (3) Guidance of the process of contour following by engaging visual data from an enhanced model of visual attention. (4) Classification of sequential tactile data using different machine learning approaches including Convolutional Neural Networks (CNN), support vector machines (SVM), and k-Nearest Neighbors (kNN). (5) Fusion of cutaneous and kinesthetic data for making decision on object classes based on the probability values obtained using the CNNs.

The paper is structured as follows. Section 2 briefly discusses the current literature on the topic. A concise explanation of the framework we are proposing is provided in Section 3. The process of tactile data acquisition is detailed in Section 4. Section 5 provides more information about the implementation of contour following. The classification of the acquired tactile data is discussed in Section 6. Results are reported and discussed in Section 7, and Section 8 concludes the work.

## 2. State-Of-The-Art

Biologically inspired cognitive architectures are a challenging research area aiming to enhance machine intelligence solutions. Many researchers in the field of robotics target visuo-haptic interaction present in humans to design more intelligent robots with capability of sensing and exploring the environment in the way humans do. Despite the vast advancements in processing and learning visual data and the huge research interest in evolving the artificial sense of touch, the optimal integration of visual and haptic information is not yet achieved.

From the psychophysics and neuroscience side, many researchers are trying to explain how the tactile and visual information contribute in humans to interpret their environment. Klatzky et al. suggest that both vision and touch rely on shape information for object recognition [3]. Other researchers study different exploratory procedures that humans apply for tactile object recognition [2] and reveal the superiority of tactile perception in presence of vision [4]. Demarais et al. [6] studied the performance of visual, tactile, and bimodal exploration of objects for both learning and testing procedures for object identification.

From the cognitive computation and robotic side, several researchers are aiming to achieve an optimal integration of visual and tactile data. Magosso [7] trained a neural network reproducing a variety of visuo-haptic interactions, including the improvement of tactile spatial resolution using visual data, resolving conflict situations, and compensating poor unisensory information by cross-modal data. Gao et al. [8] trained a deep neural network by learning both visual and haptic features, confirming the idea that the integration of visual and haptic data outperforms the case where the two sensory data features are employed separately. Burka et al. [9] designed and constructed a multimodal data acquisition system emulating human vision and touch senses. Their sensor suite includes an RGB-D vision sensor, an ego motion estimator, and contact force and contact motion detectors. Kroemer et al. [10] trained a robot using both visual and tactile data to discriminate different surfaces by touch. Calandra et al. [11] trained a deep convolutional neural network to learn regrasping policies from visuo-tactile data. The network was then used to predict the probability of success when grasping, based on a set of grasping configurations. Van Hoof et al. [12] trained a robot by reinforcement learning using an autoencoder to perform tactile manipulations based on visual and tactile data, separately. Fukuda et al. [13] designed and produced a biocompatible tactile sensor used in laparoscopic surgeries, employing both visual and tactile feedback.

On the other hand, the technology, processing, and interpretation of tactile data itself attract huge research interest. The latest advancements in technology of tactile sensors are listed in the paper of Chi et al. [1]. Liu et al. [14] took advantage of joint sparse coding to classify tactile sequences from their dissimilarities as computed by dynamic time wrapping. A sequence of tactile data acquired as palpations on a set of seven objects using a five finger robotic hands is used in Gorges et al. [15]. Song et al. [16] designed and used a tactile sensor constructed from a thin polyvinylidene fluoride film to classify different textures. In another research [17], they took advantage of a similar sensor to evaluate fabric surfaces. They trained a support vector machine over data extracted by fast Fourier-transform followed by a principal component analysis to reduce the dimensionality.

In our previous work [18], we exploited a computational model of visual attention to guide the process of tactile probing by collecting imprints sequentially from a sequence of eye fixations. In this work, using inspiration from the haptic exploration of objects by humans for object recognition [3], we follow object contours to capture sequences of tactile data. The visual information in form of a set of visually interesting points, determined by the enhanced model of visual attention presented in our previous work [19], is advantageously employed to help selecting the contours which can enhance the recognition rate. The tactile exploration of objects is also brought closer to exploration strategies in humans by adaptively changing the size of tactile sensor according to geometrical features of the object.

## 3. Framework

**Figure 1:**
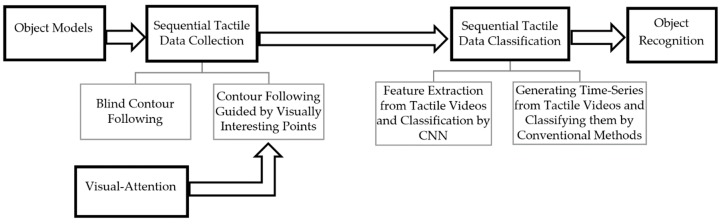
Framework for sequential tactile object recognition.

The proposed tactile object recognition framework is summarized in Figure 1. Starting from 24 object models from 8 classes, we first constructed a dataset of sequential tactile data. Relying on the contour following exploratory procedure employed by humans to perceive the general shape of objects and for object identification, in this work the sequence of tactile data is generated by following a complete contour of each model where the contour following is implemented either blindly or guided by a computational model of visual attention. The main objective is to show that the recognition rate can be improved by engaging visual data. Two different scenarios were then implemented to classify sequential data. In the first scenario, two Convolutional Neural Networks were used to learn the features from sequences of tactile images (videos of tactile imprints) and sequences of normal vectors to object surface (cutaneous cues). In the second scenario, a series of features is extracted from a set of time series extracted from tactile videos using wavelet-decomposition and then a conventional learning algorithm, such as support vector machines and K-nearest neighbors are trained and tested for object classification. Each of the mentioned time series monitors the alternation in a specific tactile feature while the tactile sensor moves along the contour of objects. These features are themselves extracted using directional contourlet transformation [20]. The rest of the paper details the process of tactile data acquisition as well as the classification.

## 4. Tactile Data Acquisition

Psychological studies on human tactile perception reveal the contribution of several forms of tactile information to be interpreted to understand a stimulus. Cutaneous data captured by skin can provide information about the temperature, vibration, roughness, and local deformations on the surface. On the other hand, kinesthetic data from muscles, joints, and bones can help make an estimation of the approximate weight and global shape of an interacted object.

In order to recognize an object by touch, roughness, and discontinuities of object’s surface (as cutaneous cues) and the finger movements to track the global shape (as kinesthetic cues) contribute together. On the other hand, contour following is the main exploratory procedure [2] that humans use to recognize an object. Accordingly, the following sections detail how, in this work, the cutaneous and kinesthetic cues are simulated and applied to reproduce the human sense of touch for robots.

### 4.1. Cutaneous Cues (Tactile Imprints)

**Figure 2:**
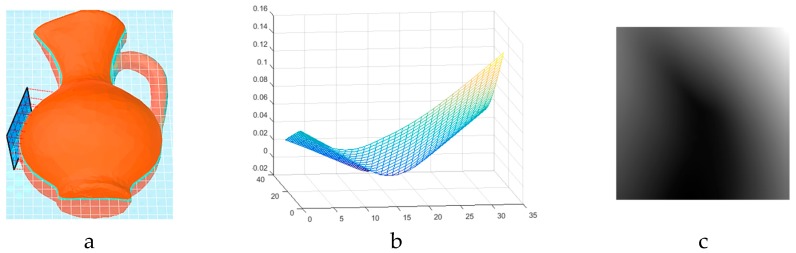
Adaptive modification of tactile sensor size.

In piezoresistive tactile sensors, which are widely accepted as a promising solution for tactile object recognition [21], the deformation in the sensor surface when subjected to an external force and in touch with an object can modify the resistance of a bridge circuit producing a proportional differential voltage. This differential voltage is then further processed to generate a tactile image. Inspired from working principle of piezoresistive sensors, we have developed a virtual tactile sensing module where the deformation measure in the surface of the tactile array is simulated as the distance between object surface and all the cells on a determined tangential plane to the object surface when the distance between the center of the plane and the object is zero. Figure 2 illustrates the simulated tactile sensor as a blue plane as well as an example of locally captured deformation profile (Figure 2b) and the produced tactile image (Figure 2c).

### 4.2. Adaptive Probing

**Figure 3:**
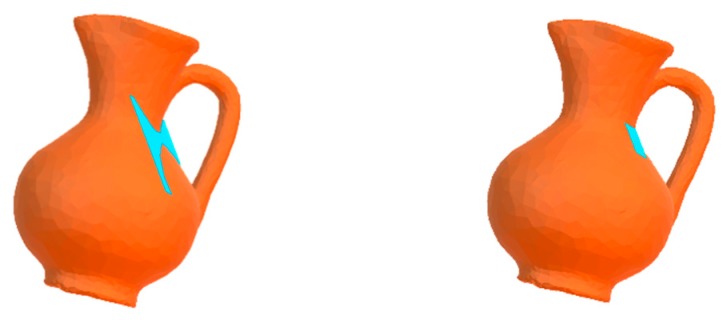
Adaptive modification of tactile sensor size.

In this work, the locations on the surface of objects, from which tactile data are captured, are previously determined using object contours (blindly or based on a model of visual attention as it will be discussed in Section 5), and the center of the sensor is considered to be positioned at these locations. Consequently, in concave surfaces, such as the example in Figure 3, the sensor surface intersects the object resulting in negative distance values between the object and sensor. Since a real rigid backing FSR sensor cannot acquire tactile data from such probing cases, we follow the haptic exploration strategy by humans, where tactile information from larger surfaces is obtained by the palm and fingertips are used for finer details and concave surfaces. Accordingly, in this work, we have adaptively adjusted the sensor size to capture the local tactile data. A real counterpart robotic hand can be designed and produced using multiple FSR arrays with different sizes placed in palm and different phalanges. Alternatively, the Barrett robotic hand [22] can be purchased equipped with tactile sensing pads across fingers (smaller pad) and palm (larger pad), which is in accordance to the use of sensors we make in this work. In order to keep the size of the tactile image consistent during the experimentations (i.e., 32×32 in the current work), the distance between the sensing points is diminished; therefore, a higher local precision is achieved since the same number of sensing elements are assigned to touch a smaller surface of the object. As such, a tactile imprint of size 32×32 is obtained for finer details of objects as well.

### 4.3. Kinesthetic Cues

As previously mentioned, kinesthetic cues can supply crucial information regarding the shape and size of the explored objects that are not perceivable by human skin. Drawing inspiration from kinesthetic cues contributing in human sense of touch, such as the angle between finger phalanges and the trajectory of finger motions when exploring an object, we have computed and used the normal vectors to the object surface in the process of object recognition. When probing an object with a real tactile sensor, the normal vectors to the surface are similarly computed and used to bring the sensor in contact with the object.

### 4.4. Sequential Tactile Data Collection

According to psychological research, when exploring an object with the hands in order to recognize it, humans tend to follow object contours to understand the global shape of the object leading to object recognition [2,3]. Relying on this biological fact, we move the tactile sensor along a complete contour of the object to simulate both cutaneous and kinesthetic cues. As a result, a video of tactile imprints for cutaneous cues is generated for each contour following, where the number of consecutive frames of the video is subsampled to 25 frames to reduce the high computational cost of data processing. Similarly, a trajectory of normal vectors and the 3D coordinate of probing locations are computed.

## 5. Contour Following

As previously mentioned, this work relies on the classification of sequential tactile data collected around contours of objects. Blind contour following and contour following guided by visually interesting points are the two strategies that are explored in this work to investigate the idea that the contour over which tactile probing takes place can play a decisive role in recognition rate.

Object contours are determined using 3D planes intersecting the object. Finding the equation of each plane, the set of points belonging both to the object and the plane, form a contour around the object. In order to find the equation of a plane in 3D space, three distinct noncolinear points are required. In this work, all the planes produced to determine probing paths are set to pass through the center of the models to avoid the selection of local contours around object extremities. As such, the center of each model is chosen as one of the three required points for formation of all planes. It is worth mentioning that such an implementation does not necessarily require visual data since supplementary tactile explorations such as the grasp stabilization method used in Regoli et al. [23] or a reinforcement learning as described in Pape et al. [24] can assist in determination of such contours. The acquisition of tactile information by exploration is both expensive in time and robot programming effort. Besides, it could lead to the acquisition of unnecessary data. All these, together with the possible advantage of visual cues in selection of more informative contours, incited us to consider the two data acquisition strategies as follows.

In the case of blind contour following, besides the central point of the model, the two other points are randomly selected from the vertices of the object model; in the case where contours are guided by the model of visual attention [19], the two other points are selected randomly from the set of visually interesting points.

**Figure 4:**
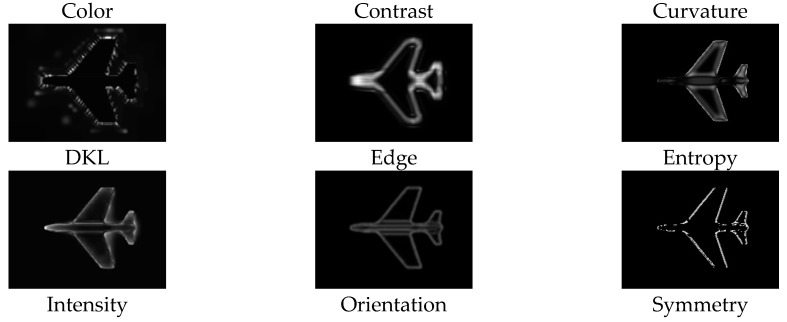

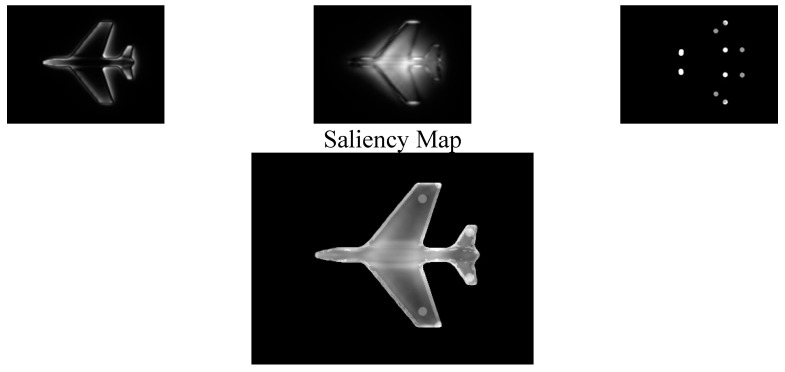
The nine channels contributing to the model of visual attention.

The computational model of visual attention presented in [19] is adopted in this work to determine visually interesting points. The model uses the virtual camera of Matlab to collect a series of images from each object such that the complete coverage of the object surface is ensured. The obtained images are then decomposed into nine channels which are believed to contribute in guidance of attentions in humans, including color opponency, DKL color space, intensity, contrast, orientation, curvature, edges, entropy, and symmetry. The contribution weight of each channel is then learned based on a set of ground-truth points identified by a set of users. The extracted visual features are finally integrated according to the computed weights as described by equation 1, where Smap is the computed saliency map, $w_{col}$,… $w_{sym}$ are the contribution weight of each feature, respectively, and $C_{col}$,… $C_{sym}$ are the feature maps illustrated in Figure 4.

(1) $$Smap=\frac{w_{col}\cdot C_{col}+w_{con}\cdot C_{con}+w_{curv}\cdot C_{curv}+w_{DKL}\cdot C_{DKL}+w_{edg}\cdot C_{edg}+w_{ent}\cdot C_{ent}+w_{int}\cdot C_{int}+w_{ori}\cdot C_{ori}+w_{sym}\cdot C_{sym}}{\sum w_{Conspicuity\;Maps}}$$

Subsequently, the brightest regions on the resulted feature map (saliency map) after setting the intensity of image background to zero are identified using a nonmaximum suppression paradigm leading to determination of visually interesting points on images. A 2D to 3D projection algorithm recuperates the 3D coordinates of each salient point, which are used in the current study to guide the determination of object contours. A detailed description about the computation of each channel as well as further details on the computational model of visual attention are available in Rouhafzay et al. [19] for interested readers.

**Figure 5:**
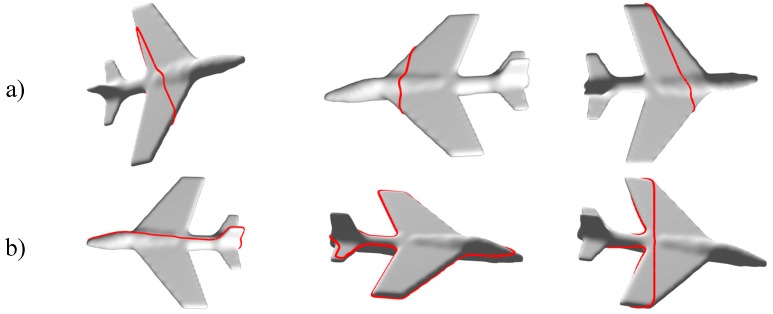
(**a**) Examples of blind contour following paths for model of Plane. (**b**) Examples of guided contour following paths by visually interesting points for model of Plane.

Figure 5a illustrates three examples of probing paths formed by random points (blind contour following), while examples of paths guided by visually interesting points are depicted in Figure 5b.

**Figure 6:**
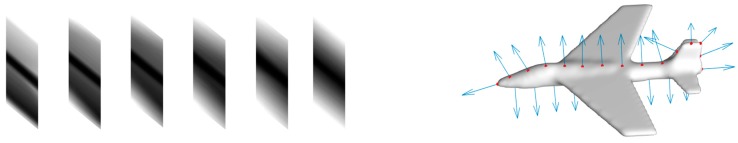
(**a**) Example of six consecutive frames of the tactile video captured from the model of Plane. (b) Example of sequence of normal vectors.

Since the number of vertices on a contour is very large and the collection of tactile data from all those vertices is neither efficient nor necessary, the obtained set of vertices is first subsampled to 25 points with equal distances between them and then cutaneous and kinesthetic data as explained in Section 4 are captured. Six of the twenty-five consecutive frames of the tactile video captured from the model of plane are depicted in Figure 6a, while Figure 6b illustrates an example of a set of normal vectors to the surface.

## 6. Sequential Tactile Data Classification

Once both cutaneous and kinesthetic cues are acquired for the objects, we implement two different approaches for object recognition by classifying the sequences of tactile data to determine if the acquired results for all techniques confirm the superiority of the model of visual attention. Convolutional neural networks allow us to feed the acquired tactile images directly; it automatically performs both feature extraction and classification of the tactile data. In order to use the two other classifiers, i.e., support vector machine and K-nearest neighbors, we need to extract ourselves the relevant features from tactile imprints and verify how these features are altered by moving the sensor around the object. This is the main distinction between the two approaches used in this paper and which are discussed in the next subsections.

### 6.1. Feature Extraction from Tactile Data and Classification by Convolutional Neural Networks

In the first approach we train two separate convolutional neural networks (CNN) to learn the features from the video of tactile images (cutaneous cues) and the sequence of normal vectors to the surface (kinesthetic cues). All tactile images captured by the virtual FSR sensor, are 32×32 grayscale images and 25 frames are considered for each exploration (contour).

The first CNN (dedicated to cutaneous cues) takes benefit from two convolution layers with 3D kernels followed by a batch normalization layer speeding up the learning process. No pooling layer is added as the size of tactile images is not so large to require down sampling. Two fully connected layers are then exploited to learn the relationship between the extracted features through the filters in the convolution layers. A SoftMax layer finally outputs the probability distribution values over predicted output classes. The network is trained for 50 epochs.

The second CNN (dedicated to cutaneous cues) has 25×3×1 data in its input layer. Thus, it can be implemented with 2D kernels in convolution layers. We set up a similar architecture for the second CNN, i.e., two convolution layers with a batch normalization in between followed by two fully connected layers, and a SoftMax layer generating the probability distribution results predicted for the output layer.

**Figure 7:**
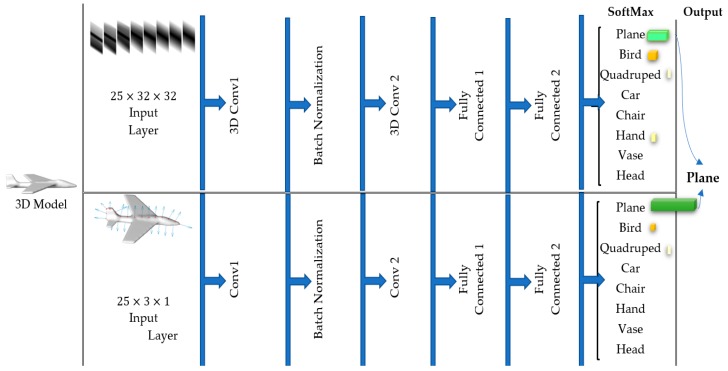
The two convolutional neural network (CNN) structures and the decision on output class.

Each of these CNNs are applied after training to tactile data captured over identical contours as a test sample and output the probabilities that the sample belongs to each of the eight object classes. The winning class has the highest probability among all. In order to integrate the results obtained by cutaneous and kinesthetic cues, for each probing sequence from the test data, we sum up the obtained probability values computed by the two CNNs for each class and choose the class with the highest probability as the winning class, as illustrated in Figure 7.

### 6.2. Feature Extraction from Time Series by Wavelet Decomposition and Classification by SVM and KNN

**Figure 8:**
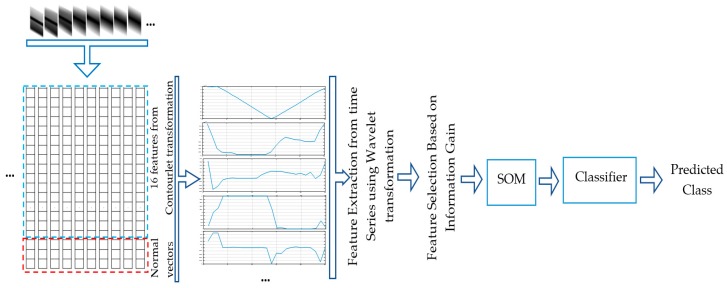
Process of object classification using conventional classifiers.

In the second approach we simplify the tactile video classification such that a set of features from videos can be directly fed into conventional classifiers. In the previous approach, we took benefit from a CNN with a 3D kernel to learn features of tactile data. Here we employ a 16 directional contourlet transformation [20] for feature extraction from each tactile imprint. As such, a feature vector of size 16 is computed as the standard deviation of each directional subband. At this point, the normal vectors to probing locations (kinesthetic cues) are added to the obtained feature vector to create a 1×19 feature vector for each probing point from the contour. The variation of each of these 19 features by moving the tactile sensor along a contour creates 19 time-series of length 25. Consequently, we exploit a 3-level wavelet decomposition for each of the 19 time-series using the Daubechies 2 wavelet, to extract features characterizing how the 19 tactile features vary when the tactile sensor moves along the object contour. Then the root mean squared (rms) value, standard deviation, and skewness of the wavelet coefficients for each level, as well as those of the sequence itself, are concatenated to produce a final feature vector. To avoid the negative effect of high dimensional data on the performance of the classifiers, a feature selection method selecting the most relevant features based on their information gain is first employed to select the most informative features and then the size of the acquired feature vector is reduced to five using a self-organizing-map. Figure 8 summarizes the data processing strategy for object recognition using a conventional classifier.

## 7. Experimental Results

**Figure 9:**
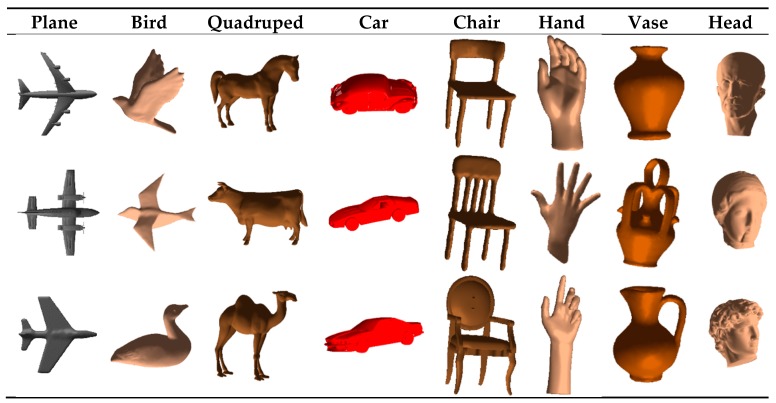
Objects used for experiments.

Figure 9 illustrates the 3D objects used in this study. Twenty-four object models belonging to eight classes are selected from a popular dataset [25]. We have applied the proposed framework to acquire sequential tactile data by blind contour following as well as using contour following paths guided by visually interesting points and the obtained sequences are classified using the previously explained approaches.

Since CNN, like any other deep learning solution, requires a large data set for training; we have collected a total of 22,800 sequences, from which 20% (4560) is used for testing and the obtained accuracy is reported in Table 1. The process of tactile data acquisition is simulated using the MATLAB programming platform and its statistics and machine learning toolbox are used for training and testing Convolutional Neural Networks.

In the case of the approach in Section 6.2, the acquired data set is first standardized using z-score before being fed into SVM and kNN. After splitting the data into 80:20 samples for developing the classifier and testing it, the conventional classifiers are trained and validated using 5-fold cross-validation. The kNN classifier takes benefit from the Euclidean distance metric to determine nearest neighbors and assigns the label of winning vote among the 10 nearest ones to test data. The support vector machine employs an RBF kernel function with γ=1, ϵ=0.5, and C=1 hyperparameters.

**Figure 10:**
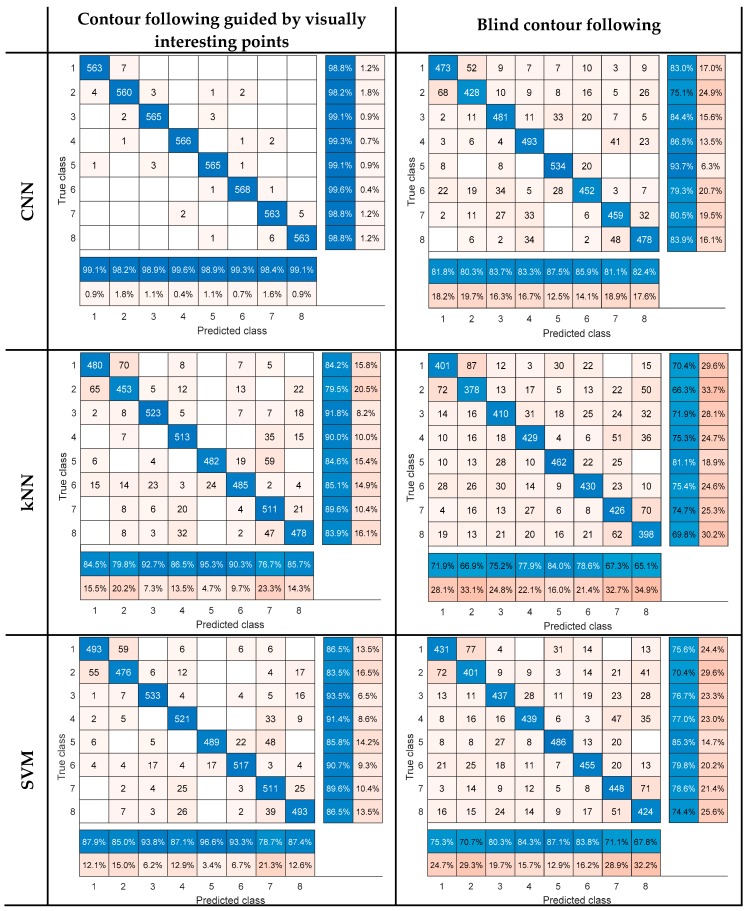
Confusion matrices.

The accuracy values for the 20% of the data kept out for testing, which includes 4560 test sequences, are reported in Table 1 for the three classifiers. Confusion matrices are also provided in Figure 10, in which the eight object classes are numbered as class one to eight in the same order in which they are presented in Figure 8.

The accuracy values confirm that the use of visually interesting points to determine object contours has a positive impact on classification accuracy for all classifiers and in the case of Convolutional Neural Networks the accuracy is improved by 15.68%. Furthermore, CNNs show a good capability in extracting and learning features from tactile data. It is worth mentioning that the random guess in our experiments is $\frac{1}{8}$ or 12.5%, and the best performance achieved in this work is 98.97%, i.e., 86.47% above the random guess.

According to the confusion matrices, the visual data helps making a cleaner discrimination among some object classes, so the confusion occurs only among the classes with more tactile similarities. This can be explained by the fact that visually interesting points lead to the selection of contours which are more informative about the object characteristics. For example, a round or oval shape contour can be followed on almost all objects if we blindly follow a local path around object extremities, while visual data guides the process by selection of different contours simplifying the object recognition process.

## 8. Conclusions

In this work, we proposed a novel framework for robotic tactile object recognition with inspiration from human tactile object exploration. The two sensing modalities in human tactile perception including kinesthetic and cutaneous cues are simulated for a set of object models and an enhanced model of visual attention intervenes to guide the process of touching. The originality of the work described in this paper is that the visual data does not directly interfere in the process of object recognition but is employed to select the most informative contours of the object that enhance the accuracy of classifiers. Two different approaches using convolutional neural networks and two other conventional classifiers (kNN and SVM) are employed to classify the tactile data for object recognition. The obtained results are compared to the case where visual data are not used confirming the fact that visual attention can improve the process of tactile data acquisition. An accuracy of 98.97% is the highest performance achieved in this study using CNN. A future application of the current work is the integration of the proposed intelligent algorithm in the decision system of a robot that can make use of its vision to select contours and then use its hand equipped with FSR tactile sensors of different sizes in finger phalanges, fingertips, and palm to touch objects and recognize them. Since for real implementation of the proposed framework the occurrence of noise may affect the performance, we will take advantage of a denoising autoencoder assisting to reconstruct the corrupted inputs.

## Figures and Tables

**Table 1:** Classification accuracy of test data.

	Contour Following Guided by Visually Interesting Points	Blind Contour Following
CNNs	98.97%	83.29%
kNN	86.07%	73.11%
SVM	88.44%	77.21%
